# Expression and Quorum Sensing Regulation of Type III Secretion System Genes of *Vibrio harveyi* during Infection of Gnotobiotic Brine Shrimp

**DOI:** 10.1371/journal.pone.0143935

**Published:** 2015-12-04

**Authors:** H. A. Darshanee Ruwandeepika, Indrani Karunasagar, Peter Bossier, Tom Defoirdt

**Affiliations:** 1 Department of Livestock Production, Faculty of Agricultural Sciences, Sabaragamuwa University of Sri Lanka, Belihuloya, Sri Lanka; 2 Centre for Science Education and Research, UNESCO MIRCEN for Medical and Marine Biotechnology, Nitte University, Mangalore, India; 3 Laboratory of Aquaculture & *Artemia* Reference Center, Ghent University, Ghent, Belgium; ContraFect Corporation, UNITED STATES

## Abstract

Type III secretion systems enable pathogens to inject their virulence factors directly into the cytoplasm of the host cells. The type III secretion system of *Vibrio harveyi*, a major pathogen of aquatic organisms and a model species in quorum sensing studies, is repressed by the quorum sensing master regulator LuxR. In this study, we found that during infection of gnotobiotic brine shrimp larvae, the expression levels of three type III secretion operons in *V*. *harveyi* increased within the first 12h after challenge and decreased again thereafter. The *in vivo* expression levels were highest in a mutant with a quorum sensing system that is locked in low cell density configuration (minimal LuxR levels) and lowest in a mutant with a quorum sensing system that is locked in the high cell density configuration (maximal LuxR levels), which is consistent with repression of type III secretion by LuxR. Remarkably, *in vivo* expression levels of the type III secretion system genes were much (> 1000 fold) higher than the *in vitro* expression levels, indicating that (currently unknown) host factors significantly induce the type III secretion system. Given the fact that type III secretion is energy-consuming, repression by the quorum sensing master regulators might be a mechanism to save energy under conditions where it does not provide an advantage to the cells.

## Introduction

*Vibrio harveyi* is a major pathogen of aquatic organisms, including shrimp and fish [1]. Several phenotypes, including motility and the production of lytic enzymes and siderophores have been associated with pathogenicity of the organism [2]. Some of the virulence factors produced by pathogenic bacteria are translocated out of the cells by dedicated secretion systems such as the type III secretion system (T3SS) [3]. T3SS enable pathogens to inject their virulence factors directly into the cytoplasm of the host cells [4]. *V*. *harveyi* contains a T3SS locus consisting of 3 adjacent operons on chromosome 1 [5] and one operon located 15 kb apart [6].

**Fig 1:**
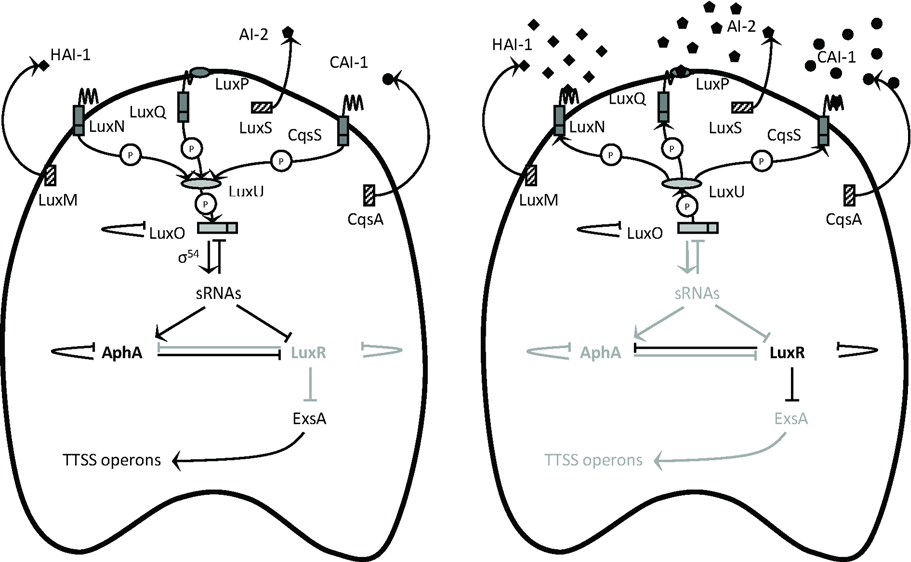
Quorum sensing in *Vibrio harveyi*. The LuxM, LuxS and CqsA enzymes synthesise the signal molecules HAI-1, AI-2 and CAI-1, respectively. These signal molecules are detected at the cell surface by the LuxN, LuxQ and CqsS two-component receptor proteins, respectively. Detection of AI-2 by LuxQ requires the periplasmic protein LuxP. **(A)** In the absence of signal molecules, the receptors autophosphorylate and transfer phosphate to LuxO via LuxU. Phosphorylation activates LuxO, which together with σ^54^ activates the production of five small regulatory RNAs (sRNAs). These sRNAs, together with the chaperone Hfq, destabilise the mRNA encoding the transcriptional regulator LuxR. Therefore, in the absence of autoinducers, the LuxR protein is not produced. LuxR is a repressor of ExsA. Hence, in the absence of signal molecules, ExsA is produced and in turn activates expression of the TTSS operons. **(B)** In the presence of high concentrations of the signal molecules, the receptor proteins switch from kinases to phosphatases, which results in dephosphorylation of LuxO. Dephosphorylated LuxO is inactive and therefore, the sRNAs are not formed and the transcriptional regulator LuxR is produced. LuxR represses ExsA, and the TTSS operons are not expressed. “P” denotes phosphotransfer.

As virulence factors are often costly metabolic products, their expression usually is controlled by regulatory mechanisms such as quorum sensing, bacterial cell-to-cell communication with small signal molecules [7,8]. *V*. *harveyi* is one of the model organisms in studies on quorum sensing in bacteria [9]. This bacterium contains a three-channel quorum sensing system, with three different types of signal molecules feeding a shared signal transduction cascade [10]. Central in the signal transduction cascade is the LuxO protein. Phosphorylated LuxO indirectly inhibits production of the transcriptional regulator protein LuxR, whereas unphosphorylated LuxO is incapable of exerting this activity because of a conformational change [11]. LuxR directly activates the *lux* operon and directly or indirectly controls many other quorum sensing target genes, including the T3SS genes (Fig 1). The *V*. *harveyi* promoters controlling the expression of the T3SS operons are repressed by the quorum sensing master regulator LuxR, i.e. expression levels are inversely related to the levels of LuxR [5,6].

Until now, most studies investigating the expression and regulation of virulence factors (including T3SS) have been performed *in vitro* using bacteria grown in (nutrient-rich) synthetic growth media. However, it is becoming increasingly clear that growth in complex natural environments (such as a host niche) contrasts with the standardized and idealized conditions in laboratory monocultures, and mechanisms that occur *in vivo* can be distinct from those that occur in simplified model systems [12,13]. Hence, studies on virulence gene expression and regulation should ideally be performed where it really matters: *in vivo* during infection of a host. An experimental procedure to measure virulence gene expression of vibrios during infection of gnotobiotic brine shrimp larvae developed earlier [14] was used here to study the *in vivo* expression of T3SS genes during infection.

## Results and Discussion

### *In vitro* expression of type III secretion system genes

The T3SS of *V*. *harveyi* ATCC BAA-1116 (recently reclassified as *V*. *campbellii* [15]) is encoded by four operons located on chromosome 1 (T3SS.1 to T3SS.4 [6]). We selected one gene from the first three operons for our analysis (T3SS.4 was not yet known to us at the moment the experiments were performed). In order to verify quorum sensing regulation of the T3SS operons, the *V*. *harveyi luxO* point mutants JAF483 (LuxO D47A) and JAF548 (LuxO D47E) were used [16]. The quorum sensing system is locked in the low cell density configuration in mutant JAF548 (henceforth denoted QS^-^) and in the high cell density configuration in mutant JAF483 (henceforth denoted QS^c^), resulting in minimal and maximal levels of the quorum sensing master regulator LuxR, respectively, irrespective of cell density or signal molecule concentration. The strains were grown to late exponential phase in marine broth and mRNA levels were measured by reverse transcription qPCR as described before [17]. mRNA levels in the wild type were set at 1 and the levels in the other strains were normalised accordingly. Consistent with repression of T3SS by the quorum sensing master regulator LuxR as reported before [5,6], expression levels of the T3SS genes were higher in the mutant with the quorum sensing system locked in low cell density configuration (QS^-^) than in the mutant with the quorum sensing system locked in high cell density configuration (QS^c^), and the wild type showed intermediate levels (S1 Fig). Van Kessel et al. recently reported that the T3SS is also repressed by a second quorum sensing master regulator, AphA, which is only produced at low cell density [18]. In view of repression by AphA, one might have expected to see higher T3SS gene expression levels in the wild type than in the QS^-^ strain since the strains were harvested at late exponential phase. Consequently, AphA will have been low or absent in the wild type, whereas the QS^-^ strain produces high levels of AphA and low levels of LuxR [18]. Apparently, repression by LuxR in the wild type was stronger than repression by AphA in the QS^-^ strain. The difference in expression levels between QS^-^ and QS^c^ was in the order of 10–20 fold (Table 1). For comparison, the differences in expression levels of the quorum sensing master regulator *luxR* and the *vhp* metalloprotease (which is induced by LuxR) were less pronounced (3–7 fold).

**Table 1:** Difference in expression of the type III secretion system genes *vopD*, *vcrD* and *vscP*, the quorum sensing master regulator gene *luxR* and the *Vibrio harveyi* metalloprotease gene *vhp* between a *luxO* mutant with the quorum sensing system locked in high cell density configuration (QS^c^) and a *luxO* mutant with the quorum sensing system locked in low cell density configuration (QS^-^), *in vitro* (in Marine Broth) and *in vivo* (in association with gnotobiotic brine shrimp larvae). The RNA polymerase A subunit (*rpoA*) mRNA was used to normalise between strains.

Gene	Difference in expression between mutants QS^c^ and QS^-^ (fold)^1^	
	*in vitro*	*in vivo* (12 h)
Type III secretion genes		
*vopD*	-20.5 (*P* < 0.05)	-8.3 (*P* < 0.01)
*vcrD*	-15.7 (*P* < 0.05)	-7.5 (*P* < 0.001)
*vscP*	-9.4 (*P* < 0.01)	-7.6 (*P* < 0.001)
Other quorum sensing-regulated genes		
*luxR* ^2^	3.3 (*P* < 0.01)	6.4 (*P* < 0.01)
*vhp* ^2^	6.8 (*P* < 0.01)	6.8 (*P* < 0.05)

^1^ negative values indicate higher expression in the QS^-^ strain than in the QS^c^ strain

^2^ Data from [17]

In addition to *V*. *harveyi*, repression of T3SS gene expression by quorum sensing has been documented for *Pseudomonas aeruginosa*, *Aeromonas hydrophila* and *Yersinia pseudotuberculosis* [19–21], whereas induction by AI-2 quorum sensing has been reported in enteropathogenic *Escherichia coli* [22]. These differences might reflect differences in the infection mechanisms employed by different pathogens.

### *In vivo* expression of type III secretion system genes during infection of gnotobiotic brine shrimp larvae

**Fig 2:**
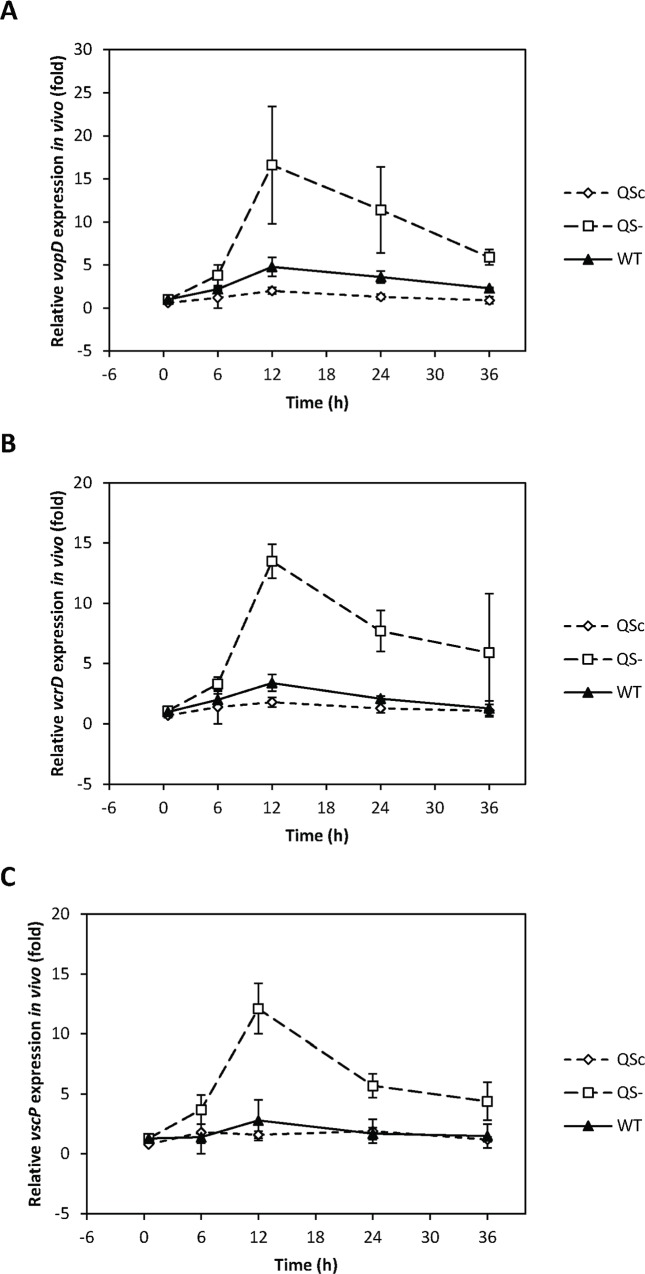
*In vivo* expression of the type III secretion system genes *vopD*, *vcrD* and *vscP*. *In vivo* expression of the type III secretion system genes *vopD* **(A)**, *vcrD* **(B)** and *vscP* **(C)** in wild type *V*. *harveyi* (WT) and mutants with the quorum sensing system locked in high cell density configuration (QS^c^) and the quorum sensing system locked in low cell density configuration (QS^-^), respectively, during infection of brine shrimp larvae. The expression in wild type *V*. *harveyi* at the 0.5h time point was set at 1 and the expression in all strains at all time points was normalised accordingly using the 2^-ΔΔCT^ method. The error bars represent the standard deviation of three independent shrimp cultures (each time based on bacterial mRNAs extracted from 500 larvae). The RNA polymerase A subunit (*rpoA*) mRNA was used to normalise between strains.

We studied the expression of the T3SS genes during infection of gnotobiotic brine shrimp larvae using a previously developed experimental procedure [14]. The expression levels of all three T3SS genes increased within the first 12h after challenge in all strains and decreased again thereafter (Fig 2). At the 12h time point, the difference in expression level between the QS^-^ strain and the QS^c^ strain was maximal and in the order of 7.5–8.5 fold, which is lower than what was observed *in vitro* (Table 1). Similar (but inverse) differences in *in vivo* expression levels of genes that are induced by quorum sensing (i.e. *luxR* and *vhp*) have been observed [17] (Table 1). Importantly, there is no difference between these strains with respect to *in vivo* cell density [23] and with respect to mRNA levels (similar C_T_ values) of the *rpoA* gene, which was used to normalise the expression data between strains. This indicates that the difference between T3SS mRNA levels during association with brine shrimp is specific and not due to differences between the strains in cell density or activity.

The *in vivo* expression levels of quorum sensing-regulated genes in the wild type and QS^-^ and QS^c^ strains are consistent with quorum sensing control, with *in vivo* expression levels of genes that are repressed by LuxR (T3SS genes) being in the order QS^c^ < WT < QS^-^, and *in vivo* expression levels of genes that are induced by LuxR (e.g. the *vhp* metalloprotease) in the order QS^c^ > WT > QS^-^. Given the fact that *in vivo* cell density is in the order of 10^9^ CFU ml^-1^ [23], AphA will have been low or even absent in the wild type *in vivo*, whereas due to the LuxO D47E point mutation, the QS^-^ strain will have produced high levels of AphA and low levels of LuxR. The fact that the QS^-^ strain showed higher T3SS gene expression levels than the wild type indicates that (similar to what we observed *in vitro*) repression by LuxR in the wild type must overrule repression by AphA in the QS^-^ strain *in vivo*. Although we currently have no data to confirm this, we hypothesise that the fourth T3SS operon will follow the same pattern as observed for the three other T3SS operons.

Mortality usually is not observed within the first 36h after challenge [23], and therefore, the peak in expression levels of the T3SS genes (as observed after 12h) occurs early during infection. Interestingly, in a previous study, a similar pattern was observed for genes that are either induced by quorum sensing or that are independent of the three-channel quorum sensing system [17]. In another study, we also observed a peak in quorum sensing activity after 12h of challenge [23]. This suggests that *Vibrio harveyi* uses a frontal attack strategy (i.e. immediate and massive attack to overwhelm the host, as defined by Merell and Falkow [24]), and that sensing of (currently unknown) host factors is integrated in the regulatory network controlling the production of the T3SS.

If the *in vivo* expression levels of the T3SS genes are normalised based on the expression level in the wild type strain grown *in vitro*, it is observed that *in vivo* expression levels in all strains are several orders of magnitude (> 1000) higher than the *in vitro* expression levels (Table 2). This indicates that sensing of the host environment largely induces the T3SS. Previous research has shown that virulence gene expression can be induced in vibrios by several features of the host environment, including (but not limited to) low iron levels [25], low oxygen levels [26], catecholamines [27], mucin, cholesterol and bile salts [28,29] and low phosphate levels [30]. Further research will be needed to investigate which of these factors (if any) is responsible for the induction of the T3SS during infection.

**Table 2:** Relative expression of the type III secretion system genes *vopD*, *vcrD* and *vscP*, the quorum sensing master regulator gene *luxR* and the *Vibrio harveyi* metalloprotease gene *vhp* in brine shrimp-associated wild type *Vibrio harveyi* and mutants with the quorum sensing system locked in high cell density configuration (QS^c^) and the quorum sensing system locked in low cell density configuration (QS^-^), respectively, after 12h challenge. The expression in wild type *Vibrio harveyi in vitro* (in Marine Broth) was set at 1 and the *in vivo* expression levels for all strains were normalised accordingly using the 2^-ΔΔCT^ method. The RNA polymerase A subunit (*rpoA*) mRNA was used as an endogenous control.

Gene	*In vivo* expression relative to wild type *in vitro* (fold)		
	Wild type	QS^c^	QS^-^
Type III secretion system genes			
*vopD*	1 938±434	812±154	6 705±2 764
*vcrD*	9 905±2 145	5 329±1 092	39 577±4 212
*vscP*	6 427±4 089	3 851±806	28 292±4 999
Other quorum sensing-regulated genes			
*luxR* ^1^	4.0±1.0	20.4±9.8	3.2±0.4
*vhp* ^1^	0.3±0.1	0.6±0.4	0.1±0.0

^1^ Data from [17]

Henke and Bassler reported before that the VopD protein (which is secreted by the T3SS) cannot be detected in cell-free culture fluids of *V*. *harveyi* mutants with a quorum sensing system locked in high cell density configuration grown *in vitro* (similar for the wild type grown to high cell density), whereas the protein was detected in mutants with a quorum sensing system locked in low cell density configuration [5]. This indicated that expression levels are very low at high cell density *in vitro*. In contrast, the high *in vivo* expression levels observed in this study suggest that when associated with a host, a functional T3SS is present in strains with an active quorum sensing system. Hence, given the fact that secretion is energy-consuming [4], repression by quorum sensing might be a mechanism to save energy by limiting the expression of the T3SS operons when they do not confer an advantage to the cells. This will result in no or very low expression of the T3SS at low cell densities (i.e. when the bacteria are in their planktonic environmental life stage) due to repression by AphA, or at high cell densities in the absence of a host (e.g. in environmental biofilms) due to repression by LuxR. It is not yet clear why there would be expression at intermediate cell densities in the absence of a host. However, it should be remarked that the expression levels in the absence of a host are much lower than those in the presence of a host (> 1000 fold in this study). The importance of quorum sensing regulation of the T3SS for infection probably is rather limited given the fact that the wild type and the QS^c^ strain are virulent, whereas the QS^-^ strain (showing the highest levels of T3SS gene expression) is not virulent [23].

## Materials and Methods

### Bacterial growth conditions

10 μl of stock cultures (maintained in 40% glycerol at -80°C) of *V*. *harveyi* strains (Table 3) [16,31] were plated onto Marine agar (Difco Laboratories, Detroit, USA) and incubated for 24 h at 28°C. Single colonies were picked from the plates and cultured in Marine broth (Difco Laboratories, Detroit, USA) at 28°C under constant agitation (150 min^-1^).

**Table 3:** *Vibrio harveyi* strains used in this study.

Strain	Relevant characteristics	References
BB120	ATCC BA-1116; wild type strain from which strains JAF483 and JAF548 were derived	[31]
JAF483	*luxO* D47A linked to Kan^R^	[16]
JAF548	*luxO* D47E linked to Kan^R^	[16]

### Axenic hatching of brine shrimp larvae

Experiments were performed with high quality hatching cysts of *Artemia franciscana* (INVE Aquaculture, Baasrode, Belgium) as described before [32]. 200 mg of cysts were hydrated in 18 ml of tap water for 1h and sterile cysts and larvae were obtained via decapsulation. Briefly, 660μl of NaOH (32%) and 10ml of NaOCl (50%) were added to the hydrated cyst suspension. The decapsulation was stopped after 2 min by adding 14 ml of Na_2_S_2_O_3_ (10 g l^-1^). Filtered (0.22μm) aeration was provided, during the reaction. The decapsulated cysts were washed with filtered (0.22μm) and autoclaved natural seawater. The cysts were resuspended in a 50 ml tube containing 30 ml filtered and autoclaved natural seawater and hatched for 24 h on a rotor (4 min^-1^) at 28°C with constant illumination (approx. 2000 lux). After 24 hours, approximately 600 animals were transferred into sterilized flasks containing 1l filtered and autoclaved natural seawater. *V*. *harveyi* strains used for the challenge were washed twice in filtered and autoclaved natural seawater and added to the cultures at 10^5^ CFU per ml of shrimp culture water. Finally, the flasks were put on the rotor and kept at 28°C. All manipulations were done in a laminar flow cabinet in order to maintain gnotobiotic conditions for cysts and larvae, basically to ensure that PCR signals are only due to the added strains.

### RNA extraction from *in vitro* grown bacteria

Each *V*. *harveyi* strain was grown to late exponential phase in three independent cultures (OD_600_ of 1). The cell density was measured spectrophotometrically (Shimadzu UV-1601, Kyoto, Japan) as absorbance at 600 nm. The cells were harvested and suspended in bacterial RNA protective reagent (Qiagen) according to the manufacturer’s instructions in order to increase the RNA stability. The pellet was stored at -80°C. RNA was extracted using the Qiagen RNeasy Mini Kit (Qiagen, Hilden, Germany) according to the manufacturer’s instructions. Extracts were subsequently treated with DNase I (Fermentas, Germany), according to producer’s guidelines to remove the remaining DNA. The RNA quantity was checked spectrophotometrically (ND-1000, V3.3.0, Thermo Fisher Scientific, USA) and adjusted to 200 ng μl^-1^ in all samples. Complete DNA degradation within the RNA samples was confirmed by subjecting DNAse-treated RNA to PCR. The RNA quality was confirmed by electrophoresis. The RNA samples were stored at -80°C for subsequent use.

### Bacterial RNA extraction from challenged brine shrimp larvae

Bacterial RNA was extracted from challenged brine shrimp as described previously [14]. Bacterial isolates used for the challenge were washed twice in filtered and autoclaved seawater. The bacterial dose was 10^5^ CFU per ml of shrimp rearing water. At the start of the challenge test, an autoclaved suspension of autoclaved LVS3 bacteria in filtered and autoclaved seawater was added as feed, equivalent to approximately 10^7^ CFU ml^-1^ rearing water. Brine shrimp larvae were sampled at 0.5, 6, 12, 24, 36 and 48 hours after addition of *V*. *harveyi* strains to the rearing water (500 larvae per sample from three independent shrimp cultures) and stored at -80°C for RNA extraction. Before the RNA extraction, the larvae were homogenized using an Eppendorf grinder (Kontes Pellet Pestle® Micro Grinders, Daigger and company, Illinois, USA) under aseptic conditions. Tissue debris of the larvae was removed using the Qiashredder (Qiagen, Hilden, Germany) apparatus to avoid clogging of the RNA extraction columns during the subsequent RNA extraction. The extraction of RNA was performed as described for the *in vitro* grown bacteria.

### Reverse transcription

Reverse transcription was performed using reverse transcriptase (Fermentas International Inc., Canada) in accordance with the manufacturer’s instructions. Briefly, a mixture of 2 μg RNA and 2 μl reverse primer solution was incubated at 70°C for 5 min and then chilled on ice. Subsequently, 8 μl of reaction mixture containing 4 μl of 5x reaction buffer (0.25 mol^-1^ Tris—HCl pH 8.3, 0.25 mol^-1^ KCl, 0.02 mol^-1^ MgCl_2,_ 0.05 mol^-1^ DTT), 2 μl of 0.01 mol^-1^ dNTP mix, 20 units of ribonuclease inhibitor (Fermentas Life Sciences), 200 units of RevertAid^TM^ H minus M MuLV reverse transcriptase (Fermentas Life Sciences) was added, the reaction mixture incubated at 42°C for 60 min followed by heating at 70°C for 10 min and then cooled to 4°C. cDNA samples were checked by PCR and stored at -20°C for further use.

### Primers used in this study

Specific primers for the T3SS genes *vopD*, *vcrD* and *vscP* were designed using the Primer 3.0 software (http://frodo.wi.mit.edu/primer3). The *rpoA* gene, which is considered to be a house keeping gene, was used as a control in the Real-time PCR [33]. Specific primers were designed based on the consensus of sequences that are deposited in GenBank. Primers used in this study are listed in Table 4.

**Table 4:** Primers used in this study.

Gene	Primer sequence	GenBank accession n°	Product size (bp)
*vopD*	F: TGA GCA ACA GGT TCT GCA AC	AY524044	198
	R: GCG ACT TCT GCC TTG ATT TC		
*vcrD*	F: TGA CAG TAC CGC TGC TCA TC	AY524044	241
	R: CCT TCG GTC ACC AAC AGT TT		
*vscP*	F: GAG ACC CTG CAG GTA TTG GA	AY524044	165
	R: CGG CTG TGA TTT ATC CGT TT		

### Real-time PCR

Real-time PCR was used to quantify the expression level of the T3SS genes. The appropriate primer concentration (300 nM) was determined for subsequent use in the real-time PCR. Dissociation curve analysis in the real-time PCR was performed for each gene to check for the amplification of untargeted fragments. Real-time PCR was performed in an ABI PRISM 7300 Fast Real Time System thermal cycler (Applied Biosystems) in a total volume of 25 μl, consisting 12.5 μl of 2 X SYBR green master mix, appropriate volumes of forward and reverse primers and 5 μl of template cDNA. The volume of each reaction mixture was adjusted to 25 μl by adding sterile RNase free water. The thermal cycling consisted of an initial activation at 50°C for 2 min, initial denaturation at 95°C for 10 min followed by 45 cycles of denaturation at 95°C for 15s, primer annealing at 58°C for 20s and elongation at 72°C for 30s. Data acquisition was performed with the 7300 SDS software (v 1.3.1) at the end of each elongation step.

### Real-time PCR data analysis

Real-time PCR data was analysed using the 2^-ΔΔCT^ method as described before [14]. The real-time PCR was validated by amplifying serial dilutions of cDNA synthesized from 1 μg of RNA isolated from bacterial samples. Serial dilutions of cDNA were amplified by real-time PCR using gene specific primers. ΔC_T_ (average C_T_ value of target—average C_T_ value of *rpoA*) was calculated for the different dilutions and plotted against the cDNA concentration. The slope of the graph was almost equal to 0 for all of the target genes. Therefore, the amplification efficiency of reference and the target genes was considered to be equal. The expression of target genes in the samples was normalized to the endogenous control *rpoA* (RNA polymerase A subunit) by calculating ΔC_T_

$$\Delta {\text{C}}_{\text{T}}={\text{C}}_{\text{T}}{,}_{\text{target}}\;-\;{\text{C}}_{\text{T}}{,}_{\text{rpo}A}$$

and expressed relative to a calibrator strain by calculating ΔΔC_T_:

$$\Delta \Delta {\text{C}}_{\text{T}}=\Delta {\text{C}}_{\text{T}}\;-\;\Delta {\text{C}}_{\text{T,calibrator}}$$

Strain JAF548 (in which the quorum sensing system is locked in low cell density configuration) was used as calibrator for the *in vitro* expression study. For the *in vivo* expression study the calibrator was the 0.5h time point of JAF548. The relative expression was then calculated as

$$\text{Relative expression}=2^{-\Delta \Delta \text{Ct}}$$

## Supporting Information

S1 Fig Expression of the type III secretion genes *vopD*, *vcrD* and *vscP* in wild type *V*. *harveyi* and quorum sensing mutants. Expression of the type III secretion genes *vopD*, *vcrD* and *vscP* in wild type *V*. *harveyi* and mutants with the quorum sensing system locked in high cell density configuration (QS^c^) and the quorum sensing system locked in low cell density configuration (QS^-^), respectively, *in vitro* after 24h incubation in Marine Broth.
(DOCX)
