# Supplementary material for: Expression and Quorum Sensing Regulation of Type III Secretion System Genes of Vibrio harveyi during Infection of Gnotobiotic Brine Shrimp
Source: PLoS One. 2015 Dec 4;10(12):e0143935. doi: 10.1371/journal.pone.0143935 (PMC4670211; doi:10.1371/journal.pone.0143935)
Supplement: S1 Fig — Expression of the type III secretion genes vopD, vcrD and vscP in wild type V. harveyi and mutants with the quorum sensing system locked in high cell density configuration (QSc) and the quorum sensing system locked in low cell density configuration (QS-), respectively, in vitro after 24h incubation in Marine Broth. (DOCX) [file pone.0143935.s001.docx]

**Expression and quorum sensing regulation of type III secretion system genes of *Vibrio harveyi* during infection of gnotobiotic brine shrimp – supporting information**

H.A. Darshanee Ruwandeepika, Indrani Karunasagar, Peter Bossier and Tom Defoirdt

**S1 Fig.** Expression of the type III secretion genes *vopD, vcrD* and *vscP* in wild type *V. harveyi* and mutants with the quorum sensing system locked in high cell density configuration (QS^c^) and the quorum sensing system locked in low cell density configuration (QS^-^), respectively, *in vitro* after 24h incubation in Marine Broth. The error bars represent the standard deviation of three independent cultures. The expression in the wild type was set at 1 and the expression in the other strains was normalised accordingly using the 2^-ΔΔCT^ method. The RNA polymerase A subunit (*rpoA*) mRNA was used as an internal control.
